# Long-term motor activity, cardiopulmonary performance and quality of life in abdominal wall defect patients

**DOI:** 10.1038/s41390-023-02900-y

**Published:** 2023-12-05

**Authors:** Christina Flucher, Jana Windhaber, Paolo Gasparella, Christoph Castellani, Sebastian Tschauner, Barbara Mittl, Vanessa Wolfschluckner, Georg Singer, Holger Till

**Affiliations:** 1 Department of Paediatric and Adolescent Surgery, Medical University of Graz, Graz, Austria; 2 Division of Paediatric Radiology, Department of Radiology, Medical University of Graz, Graz, Austria

## Abstract

**Aim:**

To assess whether patients born with an abdominal wall defect (AWD) have impaired cardiorespiratory performance capacity, motor skills, core stability or quality of life in a long-term follow up.

**Methods:**

Patients diagnosed with AWD between 2002 and 2013 were invited to participate in the study, which included clinical examination, spirometry, cardiopulmonary exercise performance testing, assessment of motor activity, ultrasound, electromyography of the abdominal wall and assessment of the Gastrointestinal Quality of Life Index (GIQLI). The results were compared to a healthy control group matched for age, sex, BMI, and physical activity levels.

**Results:**

In total, 18 AWD patients (mean age 12.6 ± 3.5 years) were included and there were no significant differences in anthopometric data compared to the control group (*n* = 18). AWD patients had a significantly lower GIQLI score (AWD mean 137.2 ± 6.8 vs. control mean 141.4 ± 4.9; *p* = 0.038) and were affected by decreased motor abilities with significantly higher Dordel-Koch-Test values (AWD median 3.54/IQR 1 vs. control median 2.8/IQR 1; *p* = 0.005).

**Conclusion:**

Follow-up examinations of AWD patients revealed decreased motor abilities and GIQLI scores while cardiopulmonary function was not different compared to healthy controls. The clinical impact of these findings remains to be elucidated.

**Impact:**

- Clinical examination, assessment of the gastrointestinal quality of life, sport medical testing, electromyography and abdominal wall ultrasound were performed in patients with congenital abdominal wall defect and compared to an age and sex matched healthy control group.
- Results of spirometry and spiroergometry, ultrasound or electromyography did not significantly differ between the groups.
- Significantly decreased locomotor function and gastrointestinal quality of life were found in patients with abdominal wall defect. However, the clinical impact of these findings remains to be elucidated.

## Introduction

Gastroschisis (GS) and omphalocele (OC) represent the two most common congenital abdominal wall defects (AWDs) occurring with an incidence of 4.5 of 10,000 live births and 0.6–4.8 of 10,000 live births, respectively. As opposed to other congenital malformations, a rising incidence of GS has been shown in the recent years.^1–4^

GS is usually a solitary anomaly and the outcome is related to the underlying integrity of the prolapsed bowel loops. In contrary, OC is frequently associated with other malformations such as chromosomal or cardiac defects or syndromes like pentalogy of Cantrell and Beckwith Wiedemann syndrome.^5^ Both forms of AWDs necessitate early postnatal surgical intervention, mostly in one or two stages, as well as support at a neonatal or surgical intensive care unit. Mortality is lower in GS patients than in these born with OC mostly due to associated anomalies.^6^

The literature concerning long-term outcome and quality of life (QOL) of patients suffering from AWDs is scarce. Some publications have reported long-term complications like redo surgical procedures because of fascial gaps or umbilical and incisional hernias.^7,8^ Furthermore, stool irregularities, abdominal pain and several hospital admissions due to ileus or sub-ileus have been described.^7^ Additionally, half of the patients are unsatisfied with the cosmetic result.^9–11^ Other studies, however, have revealed that children born with an AWD have the same QOL compared with the healthy population.^11^

Recent studies have revealed a decreased cardiopulmonary performance capacity (CPC) in patients born with congenital malformations such as anorectal malformations or esophageal atresia.^12,13^ However, only one report describes the cardiopulmonary outcome in patients with large AWDs (GS > 4 cm, OC > 6 cm) including 18 participants. The authors reported that patients operated on for AWD at birth exhibit a normal cardiorespiratory function.^14^

The trunk musculature including the transversus abdominis, internal and external oblique and rectus abdominis muscles are important for core and especially for spine stability.^15^ However, the abdominal musculature of AWD patients compared to a healthy control group has not been studied yet.

Therefore, it was the aim of this single-center observational long-term outcome case-control pilot study to assess whether patients born with AWDs have a decreased CPC, impaired motor skills and QOL compared to healthy controls.

## Patients and methods

All patients born between 2002 and 2013 (aged between 6 and 18 years) diagnosed with an AWD and treated at our Department were invited to participate in a prospective study consisting of clinical examination, spirometry, cardiopulmonary exercise performance testing (CPET), assessment of the motor activity, ultrasound and electromyography of the abdominal wall, stance and gait analyses and questionnaires for gastrointestinal QOL. All measurements were performed in one day. Patients with hemodynamic relevant cardiac disease or mental disorders were excluded.

According to the literature, we classified GS as complicated if patients suffered from concomitant intestinal atresia, volvulus, necrosis or perforation.^16–18^ Giant OC was defined as a defect larger than 5 cm.^19,20^

The results were compared to a healthy age-, sex-, BMI- and physical activity level-matched control group recruited from friends and family of the Department´s employees.

This study was performed according to the declaration of Helsinki. All patients and controls and/or their legal guardians gave informed written consent. This study was approved by the institutional review board (EK 32–231 ex 19/20). All measurements were performed between May 2020 and June 2021.

### Anthropometric data

Body height (BH) and weight (BW) were measured and the body mass index (BMI) calculated. Segmental multi-frequency impedance spectroscopy (Combyn^TM^ ECG, Academic Technologies at the Institute of Cardiovascular Medicine GmbH, Graz, Austria) was used to measure appendicular muscle mass and total body fat (TBF) as previously described in the literature.^21^ Cardiac arrhythmias were excluded with a 12-lead resting electrocardiography (ECG) and non-invasive blood pressure (NIBP) measurement at rest was performed.

Participants were asked to rate their physical activity levels according to four groups (“daily”, “several times a week”, “once per week” or “once per month”).

### Spirometry

Lung function was measured by small spirometry (Oxycon Pro^®^ Carl Reiner GmbH, Vienna, Austria) at rest and following exercise. Maximum vital capacity (VC_max_) and the forced expiratory volume in 1 s (FEV 1) were assessed.

VC_max_ was expressed as observed and corrected according to the expected maximum vital capacity over age and sex. The Tiffeneau index was calculated as FEV 1/VC_max_. A restrictive ventilation disorder was defined as a predominantly decreased VC_max_ and an obstructive ventilation disorder as a decreased Tiffeneau index.^22^

### Cardiopulmonary exercise performance testing (CPET)

CPET with a bicycle ergometer (Excalibur Sport^®^, Lode B.V., Groningen, The Netherlands) and the spirometer in an upright position was used to measure cardiopulmonary exercise performance. A stepwise load increase protocol, specified for sex and age, was used as published before.^23^ The spiroergometry was continued to subjective exhaustion or until the participants were unable to maintain the required pedaling speed (cadence) of more than 60 revolutions per minute (rpm). A three minutes “cool down” of slow pedaling (60 rpm) with the same workload as at the beginning of the test followed the exercise phase.

Twelve-lead ECG (Cardinal Health^TM^ electrocardiography, Dublin, Ireland) measured Heartrate (HR) and finger pulse oximeter (Habel Medizintechnik^®^, Vienna, Austria) assessed oxygen saturation continuously during the whole exercise.

At the end of each step and after the cool down lactate levels were determined by earlobe sampling of 20 µl blood per measurement to heparinized capillaries before the test (enzymatically amperometric measurement with a Biosen C_line^®^ (EKF Diagnostics for life, Cardiff, UK)).

Respiratory parameters including the oxygen uptake (VO_2_), the oxygen pulse (O_2_/HR), the respiratory equivalent for oxygen (EQO_2_), the breathing reserve (BR) and the respiratory exchange ratio (RER) were assessed.^23^

Relative performance capacity was calculated from the achieved maximal wattage in relation to age and sex-specific standard values.^24^ The peak oxygen uptake (peak VO_2_) was defined as the average VO_2_ over the last 30 seconds prior to subjective exhaustion and was expressed in ml/kg/min. A RER > 1.10 was used as criterion to determine that the peak VO_2_ reflects a peak physiological workload.^25^

### Assessment of motor abilities

The Dordel-Koch-Test (DKT) was used to assess motor abilities (flexibility, coordinative and conditional skills).^26^ The tests consists of seven established and validated items: lateral jumping, sit and reach, sit-ups, long stand jump, one-legged stand, push-ups and 6-min-run and allows a quick and differentiated evaluation of motor performance among all basic motor skills.^26^ In the present study, the endurance was tested with a spiroergometry instead of a 6-min-run. The indicated grades 1 to 6 correspond to a school grading system with lower values indicating better performance.^26^

### Electromyography (EMG) of the abdominal wall & gait and stance analysis

Eight sensors as shown in Supplementary Fig. 1 were fixed to the abdominal wall (Ultium^®^ Wireless Surface EMG, Velamed GmbH with Ultium^®^ EMG Sensor, Velamed GmbH and Noraxon MR 3.14, Cologne, Germany). Afterwards, patients had to perform eight exercises to measure activity of the M. rectus abdominis (RA), M. obliquus externus (OE)/internus (OI) and M. transversus abdominis (TA). Exercises are shown in Supplementary Fig. 2. The EMG amplitude of each muscle and exercise was normalized to the amplitude observed in isometric maximum voluntary contraction (MVC) for each muscle. The neural activity was expressed as percentage of the MVC for each muscle.^27^

A floor-based foot pressure measurement device (Zebris (F64x240x3), Velamed GmbH, Noraxon MR 3.14, Cologne, Germany) was used for gait and stance analyses in order to measure core stability. For stance analyses, the trajectory of the center of pressure (COP) was assessed while ordinary relaxed stand and Matthias’ Arm-Raising Test on the plate. Gait analyses were performed by walking over the plate for 3 min. Exercises are shown in Supplementary Fig. 3. The whole examination was filmed for later analyses (Logitech HD Pro Webcam C920, Logitech Europe S.A., Lausanne, Switzerland).

### Ultrasound of the abdominal wall

**Fig. 1:**
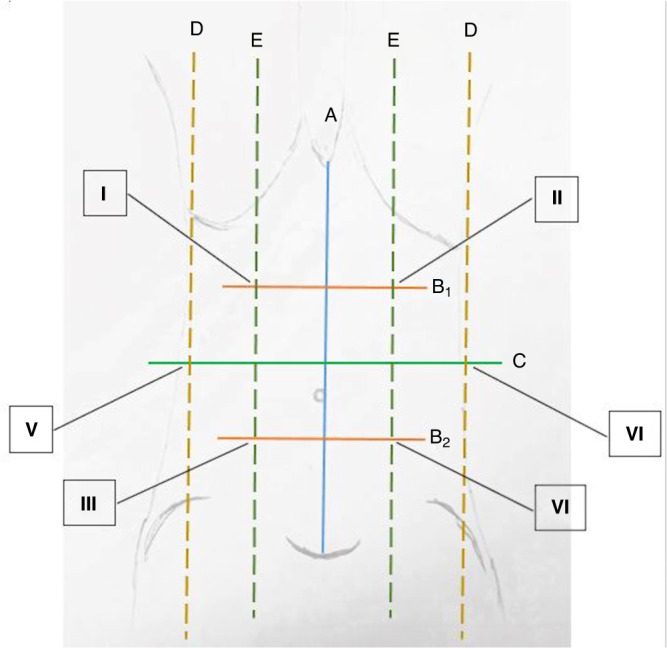
Ultrasound of the abdominal wall. Schematic drawing of the positions Q6for sonography. Schematic drawing of the positions for sonography of the abdominal wall.

Ultrasound of the abdominal wall (GE Healthcare Vivid S5 Ultrasound Machine/GE Healthcare 12L-RS probe, Solingen, Germany) was performed to assess the thickness of the four muscles (RA/OE/OI/TA). A protocol was established as shown in Fig. 1. First, the distance between the xiphoid and the symphysis was halved (C), divided in thirds (B1/B2) and marked with a skin marker. Then, the medioclavicular and anterior axillar lines were marked. The rectus abdominis muscle was measured at its thickest point in the sagittal axis in B1, B2 and C. At the crossing I-VI the OE, OI and TA were measured in the sagittal axis. All tests were performed by the same examiner (CF).

### Quality of life and cosmetic satisfaction

The Gastrointestinal Quality of Life Index (GIQLI) was assessed in all participants. This index is a tool to assess the QOL, specifically for patients with gastrointestinal disorders. In total, the questionnaire consists of 36 items answered by the patient. The questions can be separated in five dimensions: core symptoms, physical items, psychological items, social items and disease- specific items. Each question is scored from 0–4 (Likert Scale) whereas 4 is the most favorable outcome. The scoring system ranges from 0 to 144 with higher scores describing a better QoL.^28^

Moreover, patients had to rate their stool consistency according to the Bristol Stool scale, a validated tool that has been used in children before.^29^

All patients were asked if they suffer from backpain and/or gastroesophageal reflux. If yes, the patients were asked to quantify their occurrence (“never”, “once per month”, “once per week” or “daily”).

The POSAS (Patient and Observer Scar Assessment Scale) as a well validated tool to assess the quality of the scar and cosmetic satisfaction was used in this study.^30, 31^ Patients (PSAS) and the Observer (OSAS) had to assess the scar on the abdominal wall. The scoring system ranges from 6–60 with lower scores describing a better cosmetic result. In addition, both had to give an overall opinion of the scar (1–10). Lower scores mean higher quality or satisfaction with the scar.

A clinical examination of the abdomen was performed to identify length, width and position of the scar. It was checked if an umbilicus, hypertrophic scar, scar hardening, additional scars and visible stichtes were present. All examinations were performed by the same person (CF).

### Statistical analysis

Data were entered in an Excel 2016^®^ spreadsheet. For statistical analysis SPSS Statistics 27^©^ (IBM Corp. Released 2020. IBM SPSS Statistics for Windows, Version 27.0. Armonk, NY: IBM Corp) was used. Data were tested for normal distribution applying the Kolmogorow-Smirnow test. In case of normal distribution, data are depicted as mean and standard deviation and a two-sided, unpaired *t* test was used for statistical group comparison between AWD patients and controls. If no normal distribution was found, data are displayed as median and interquartile range (IQR) and group comparisons were performed with Mann–Whitney-U tests. Pearson tests were used to analyze correlations between metric parameters and Spearman tests for correlation analysis between ordinal and metric data. The Fishers exact test was used for group comparison in case of categorical data. Statistical significance was defined as *p* < 0.05.

## Results

**Fig. 2:**
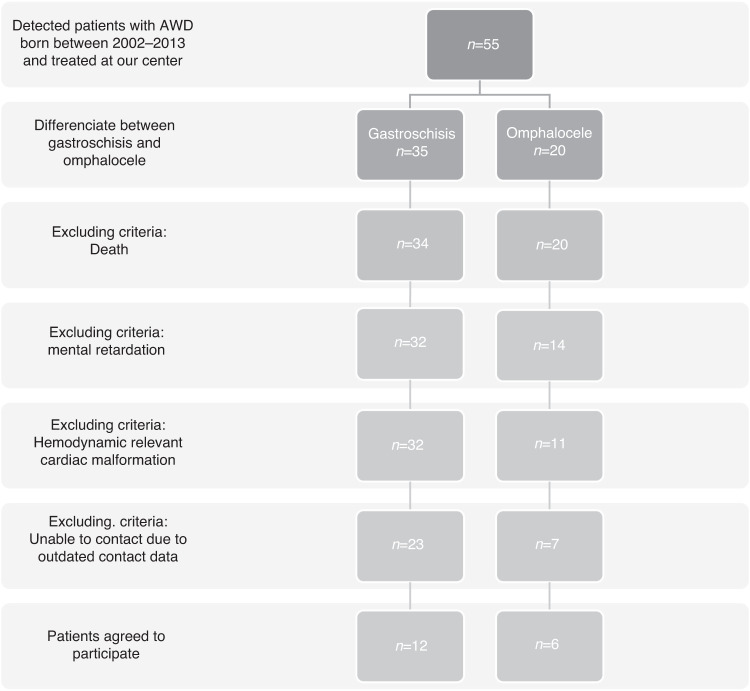
CONSORT diagram of the included and excluded patients.

Out of 43 eligible patients treated with an AWD in the respective period, we were able to contact 30 patients and 18 agreed to participate in the study. A detailed CONSORT diagram of the included and excluded patients is provided in Fig. 2.

### AWD group

The mean age of the patients (7 males, 11 females) was 12.6 ± 3.5 years (range: 7–18). Table 1 shows the different types of AWD (GS: *n* = 12, OC: *n* = 6) and their treatment.

**Table 1:** Clinical data of 18 AWD patients including type of AWD, surgical procedure, associated malformations, complications and total number of operative procedures affecting the abdominal wall.

ID	Age [years]	Sex	Type of AWD	Classification	Associated malformations	Procedure	Complications	Total number of procedures
1	7	f	gastroschisis	uncomplicated	–	PC	UH (22 mo)	2
2	7	f	gastroschisis	complicated	colon atresia	PC + colostomy		2
3	8	f	gastroschisis	uncomplicated	–	PC		1
4	10	f	gastroschisis	uncomplicated	–	PC		1
5	11	f	gastroschisis	uncomplicated	–	PC	IDA (1mo)	3
6	15	f	gastroschisis	uncomplicated	–	PC	IDA and MV (6yo)	2
7	15	f	omphalocele	small	–	PC		1
8	16	f	omphalocele	small	–	PC		1
9	16	f	gastroschisis	uncomplicated	–	PC	UH (25 mo)	2
10	18	f	omphalocele	small	ileum atresia, combined immune- deficiency, intractable diarrhea	PC + ileostomy	IDA (12yo)	5
11	18	f	gastroschisis	uncomplicated	–	PC		1
12	8	m	omphalocele	small	–	PC		1
13	12	m	gastroschisis	uncomplicated	–	SC (silo)		2
14	13	m	omphalocele	giant	pulmonary hypo- and dysplasia, ASD, eventration of the right diaphragm	PC		5
15	13	m	gastroschisis	uncomplicated	–	PC		1
16	13	m	gastroschisis	uncomplicated	–	PC	MV/IDA (1wo/3mo)	4
17	13	m	gastroschisis	uncomplicated	–	PC	UH, DR (23 mo)	2
18	13	m	omphalocele	giant	pulmonary hypoplasia, ASD	SC (silo+patch)	PI (2mo)	4

*ASD* atrial septal defect, *PC* primary closure, *SC* secondary closure, *UH* umbilical hernia, *IDA* ileus due to adhesions, *MV* midgut volvulus, *DR* diastasis recti,

*PI* patch infection.

One patient (8%) with GS had a congenital colonic atresia as an associated malformation and was classified as complicated GS in comparison to eleven (92%) uncomplicated GS cases. Two OC cases (33%) were defined as a giant OC and four as small OC (67%). In one small OC, an ileum atresia had been treated. The detailed associated malformations are listed in Table 1.

In fourteen patients (77.8%) primary closure within the first 24 h could be achieved. Two children were born with an intestinal atresia necessitating creation of a stoma and primary closure of the AWD. In one case it was necessary to perform a delayed closure using a spring-loaded silo and in one case secondary closure was performed with a patch following primary application of a silo (Table 1).

Overall, the mean number of procedures affecting the abdominal wall was 2.2 ± 1.4. The maximum number was five surgical procedures in one patient. There was no significant difference in the number of surgeries between OC and GS patients (OC mean 2.8 ± 2 vs GS mean 1.9 ± 0.9; *p* = 0.334; unpaired two-sided *t* test).

In eight patients postoperative complications, necessitating operative procedures within the first twelve years of life, were detected. In three of these patients (37.5%) post-operative complications occurred in the first year of life: one patient suffered from midgut volvulus and an ileus due to adhesions, another one from an ileus due to adhesions and in one case a patch infection after two months was recorded.

Furthermore, in three other cases an umbilical hernia was detected and repaired (at an age of 1, 1 and 2 years, respectively). One case of a midgut volvulus and ileus due to adhesions at the age of six years and one more patient with an ileus due to adhesions at the age of twelve was reported.

### Control group

The mean age of the age-, sex- and BMI-matched healthy control group (7 males, 11 females) was 12.3 ± 3.3 years (range: 7–17 years). There was no statistically significant difference regarding patient age between AWD and control groups (*p* = 0.798; unpaired two-sided *t* test).

### Anthropometric data and laboratory results

Height, weight, BMI, body fat percentage and muscle mass were not statistically significant different between the study and the control group (Table 2). Likewise, no significant differences in physical activity levels could be found.

**Table 2:** Anthropometric data, results of spirometry and spiroergometry of AWD patients and age-, sex- and BMI-matched controls. All data are displayed as mean ± standard deviation unless otherwise specified.

	AWD patients [*n* = 18]	Controls [*n* = 18]	*p* value
Age	12.6 ± 3.5	12.3 ± 3.3	0.798^a^
Anthropometry			
Height [m]	1.59 (0.28)^a^	1.60 (0.24)^a^	0.983^**^
Body Weight [kg]	48.1 ± 16.7	49.7 ± 16.3	0.785^*^
BMI	19.4 ± 4.4	19.7 ± 4.0	0.787^*^
Body Fat [%]	13.8 (18.1)^a^	10.8 (20.0)^a^	0.888^**^
Muscle Mass [kg/Height²]^b^	6.3 ± 1.3	6.2 ± 1.5	0.943^*^
Physical Activity Level^c^	2 (1)^a^	2 (0)^a^	0.521^**^
Spirometry			
VC_max_ [L]	2.9 ± 1.0	3.1 ± 1.1	0.558^*^
Tiffeneau Index [%]	86.4 ± 7.6	86.5 ± 7.6	0.965^*^
Spiroergometry	***n*** = **13**^**d**^	***n*** = **13**^**d**^	
Relative Performance [%]	97.9 ± 24.3	98.0 ± 20.3	0.986^*^
RER	1.16 ± 0.08	1.14 ± 0.11	0.600^*^
Peak VO_2_ [ml/kg/min]	40.3 (11.0)^a^	39.7 (17.0)^a^	0.555^*^
O_2_/HR [ml]	11.0 (4.0)^a^	11.2 (4.0)^a^	0.751^**^
EQO_2_	21.7 ± 3.1	19.7 ± 2.4	0.071^*^
BR FEV%	10.5 ± 10.8	14.2 ± 17.7	0.510^*^
QoL			
GIQLI	137.2 ± 6.8	141.4 ± 4.9	0.038^*^
Bristol Stool scale	4 (1)^a^	3 (1)^a^	0.111^**^

Statistically significant *p*-values are in bold.

*AWD* abdominal wall defect, *m* meter, *kg* kilogram, *VC*_max_ maximum vital capacity, *RER* respiratory exchange ratio, *peak VO*_2_ peak oxygen uptake, *O*_*2*_*/HR* oxygen pulse, *EQO*_2_ respiratory equivalent for oxygen, *BR* breathing reserve, *FEV*% Forced Expiratory Volume, *QoL* quality of life, *GIQLI* gastrointestinal quality of life index.

^*^Unpaired *t* test, ^**^ Mann–Whitney-U test.

^a^Median (IQR).

^b^*n* = 14, it was not possible to detect muscle mass in 4 cases.

^c^Physical Activity Level (once per month=0, once per week=1 several times a week=2, daily=3).

^d^Five patients were not able to perform spiroergometry because they were too short for ergometry.

### Spirometry

Neither VC_max_ nor Tiffeneau index was significantly different between the control group and AWD patients (Table 2). Likewise, comparisons of spirometry values revealed no significant differences between patients with GS and OC. Detailed data is shown in Supplementary Table 1.

Two patients in the AWD group showed a restrictive ventilation disorder. These two were born with giant OC and lung hypoplasia (ID 18) and additional lung dysplasia in one case (ID 14). In the control group, four probands showed an obstructive and one a restrictive ventilation disorder.

### Cardiopulmonary exercise performance

RER Value (AWD mean 1.16 ± 0.08 vs. controls mean 1.14 ± 0.11) did not differ significantly between the two groups (*p* = 0.600; unpaired *t* test,*)*.

Spiroergometry was not significantly different regarding relative performance capacity, peak VO_2_, O_2_/HR, EQO_2_ and BR (Table 2).

Comparing patients with GS and OC no statistically significant differences in spiroergometry were seen. Detailed data is shown in Supplementary Table 1.

There was no significant difference of the relative performance capacity between the group of uncomplicated and complicated GS as well as between small and giant OC (data not shown).

### Assessment of motor abilities

The Dordel-Koch Test (DKT) revealed significantly decreased values in the AWD group (*p* = 0.005*; unpaired two-sided t test*). In detail, AWD patients had significantly lower values concerning lateral jumping and sit-ups (Table 3). However, no statistically significant differences between patients with GS and OC were found (Supplementary Table 1).There was no significant correlation between DKT and BMI (r = 0.213, *p* = 0.213; *Spearman test*), muscle mass (r = 0.241, *p* = 0.183; *Spearman test*), total body fat percentage (r = 0.171, *p* = 0.318; *Spearman test*) or relative performance capacity (r = −0.001, *p* = 0.994; *Spearman test*). However, we found a statistically significant negative correlation between DKT and physical activity level (r = −0.379, *p* = 0.022; Spearman test). There was no significant difference of the DKT results comparing uncomplicated and complicated GS as well as small and giant OC (data not shown).

**Table 3:** Dordel-Koch-Test (DKT) of AWD patients vs. age-, sex- and BMI-matched controls. All data are displayed as median and interquartile range; statistical comparisons were performed with the Mann-Whitney-U test.

	AWD (*n* = 18)	Controls (*n* = 18)	*p* value
Lateral Jumping	4 (1)	3 (2)	0.037
Sit and Reach	4 (2)	3 (1)	0.171
Sit-Ups	4 (1)	3 (2)	0.003
Long Stand Jump	4.5 (1)	4 (1)	0.068
One-legged Stand	1 (3)	1 (0)	0.252
Push-Ups	3 (1)	2 (1)	0.111
DKT	3.4 (1)	2.8 (1)	0.005

### Gait and stance analyses and electromyography (EMG) of the abdominal wall

Except for cadence and stride time there were no statistically significant differences in stance and gait analyses between the AWD and the control group (Table 4).

**Table 4:** Stance and gait analyses of AWD patients vs. age-, sex- and BMI-matched controls; all data are displayed as median and interquartile range; statistical comparisons were performed with the Mann-Whitney-U test.

	AWD (*n* = 18)	Controls (*n* = 18)	*p* value
Stance Analyses			
Ordinary relaxed stand (ORS)			
Test duration [sec]	30.0 (4.5)	29.4 (1.97)	0.590
COP sway ellipse (cm^2^)	2.1 (6.4)	4.6 (6.1)	0.483
total COP path [mm]	302.0 (308.8)	372.0 (386.8)	0.303
COP average speed [mm/s]	9.5 (9.3)	13.0 (13.0)	0.369
Matthias’ Arm-Raising Test (MART)			
Test duration [sec]	29.2 (4.7)	28.9 (4.6)	0.732
COP sway ellipse (cm^2^)	2.6 (6.0)	5.7 (6.1)	0.232
total COP path [mm]	299.0 (286.0)	369.0 (386.3)	0.184
COP average speed [mm/s]	10.0 (10.0)	13.5 (12.8)	0.153
Change in COP ORS / MART (cm^2^)	27.0 (239.0)	−81.5 (227.0)	0.163
Gait Analysis			
Stance time [%]	63.1 (2.8)	63.2 (1.6)	0.481
Single support [%]	37.0 (2.8)	36.8 (1.6)	0.481
Gait line [mm]	209.5 (42.0)	208.5 (27.9)	0.462
Cadence [steps/min]	112.0 (19.0)	104.0 (11.8)	**0.031**
Gait speed [cm/s]	3.7 (0.7)	3. 5 (0.7)	0.389
Step length [cm]	55.0 (11.3)	55.5 (9.5)	0.770
Step time [ms]	535.5 (75.0)	592.5 (75.9)	0.086
Stride length [cm]	112.0 (15.0)	114.5 (28.0)	0.782
Stride time [ms]	1073.0 (151.0)	1154.0 (136.0)	**0.031**

Statistically significant *p*-values are in bold.

*COP* center of pressure.

The EMG amplitude of each muscle (RA, OI, OE, TA) and exercise showed no significant differences between the AWD and control group as well as between GS and OC patients (data not shown).

There was no statistical difference in duration (seconds) of performing the exercise “plank” (AWD mean 20.7 ± 14.7 vs. controls mean 21.3 ± 10.5; *p* = 0.981; *unpaired t test*,) and “lift and hold legs” (AWD median 14/ IQR 16 vs. controls median 10.5/ IQR 9; *p* = 0.126; *Mann–Whitney-U Test*). Exercises are shown in detail in Supplementary Fig. 2. Four patients of the AWD group were not able to perform the exercise “plank”. Two controls and one patient with AWD were not able to perform the exercise “lift and hold legs”.

### Ultrasound of the abdominal wall

Thickness of the abdominal wall muscles (RA/OE/OI/TA) showed no statistical difference between AWD and control patients (Table 5). Likewise, GS and OC patients did not have significant differences. Details are shown in Supplementary Table 2. There was also no statistical difference between measurements on the right and the left side of the abdomen. Detailed values are presented in Supplementary Table 3.

**Table 5:** Ultrasound of the abdominal wall of AWD patients vs. age-, sex- and BM-matched controls. All data are displayed as median and interquartile range; statistical comparisons were performed with the Mann-Whitney-U test.

	AWD (*n* = 18)	Controls (*n* = 18)	*p* value
I OE	3.4 (4.0)	2.5 (0.9)	0.135
II OE	2.9 (1.7)	2.2 (1.5)	0.211
III OE	3.8 (2.3)	3.1 (1.8)	0.203
IV OE	4.2 (2.5)	3.2 (1.8)	0.192
V OE	5.0 (3.9)	6.2 (2.0)	0.239
VI OE	5.5 (3.7)	6.5 (2.2)	0.696
I OI	3.1 (1.7)	3.8 (1.5)	0.057
II OI	3.5 (2.0)	3.2 (1.8)	0.279
III OI	5.1 (2.4)	4.7 (2.1)	0.839
IV OI	4.6 (2.9)	4.8 (2.6)	0.443
V OI	6.3 (3.5)	6.7 (3.0)	0.372
VI OI	7.1 (2.9)	6.1 (3.0)	0.521
I TA	2.5 (1.2)	2.6 (0.9)	0.287
II TA	2.6 (1.4)	2.7 (1.2)	0.660
III TA	2.2 (2.1)	2.6 (1.6)	0.152
IV TA	2.4 (1.6)	3.0 (1.1)	0.171
V TA	3.4 (2.2)	3.1 (1.6)	0.888
VI TA	3.3 (1.9)	3.4 (1.8)	0.963
B_1_ RA right	8.0 (4.8)	8.3 (3.2)	0.782
B_1_ RA left	8.7 (4.1)	7.7 (5.1)	0.546
B_2_ RA right	8.6 (3.6)	8.6 (4.1)	0.839
B_2_ RA left	9.6 (4.3)	8.8 (3.9)	0.462
C RA right	8.7 (3.6)	8.5 (4.7)	0.963
C RA left	8.9 (3.5)	8.6 (4.6)	0.815

### Evaluation of cosmetic satisfaction and quality of life

GIQLl was significantly different between the AWD and control group (AWD mean 137.2 ± 6.8 vs. controls mean 141.4 ± 4.9, *p* = 0.038; *unpaired two-sided t-test*) but not between GS and OC (GS mean 137.5 ± 7.7 vs. OC mean 136.5 ± 5.2, *p* = 0.749; *unpaired two-sided t test*). However, there were no significant differences in GIQLI between the group of uncomplicated and complicated GS as well as between small and giant OCs (data not shown). There was no significant correlation between the number of surgeries and GIQLI (r = 0.154, *p* = 0.542; *Spearman test*).

While in the AWD group six patients suffered from back pain (*n* = 5 several times a month and *n* = 1 several times a week), four patients of the control groups reported back pain (*n* = 2 several times a month and *n* = 2 several times a week). These differences were not significantly different (*p* = 0.354; *Fisher´s Exact test*).

The rate of gastroesophageal reflux was not significantly different between AWD patients (*n* = 1 several times a month and *n* = 1 daily) and controls (*n* = 1 several times a month) (*p* = 0.500; *Fisher´s Exact test*).

16 patients (94%) indicated a normal stool frequency as “every other day to twice a day” and one (6%) “more often” in the AWD group. 13 patients (76%) of control group rated their stool frequency as “every other day to twice a day” and four (24%) as “more often”. In both groups most participants classified their stool consistency as Bristol Stool Scale (BSS) type 3 or 4 (AWD: type 2 n = 1 (6%), type 3 *n* = 5 (27%), type 4 *n* = 11 (61%), type 6 *n* = 1 (6%) and controls: type 3 *n* = 12 (67%), type 4 *n* = 6 (33%)). There was no significant difference between AWD group and controls concerning stool frequency or consistency.

In nine cases (*n* = 6 GS; *n* = 3 OC) the umbilicus was the only visible scar following abdominal wall closure. Seven patients (*n* = 4 GS; *n* = 3 OC) had a horizontal and one a vertical main scar. Six patients had additional scars on their abdominal wall (*n* = 3 GS; *n* = 3 OC) and in twelve cases stiches were visible as scars (*n* = 10 GS; *n* = 2 OC).

Cosmetic satisfaction was good, as patients rated their overall opinion with 3.9 ± 2.8 and the observer overall opinion was 2.7 ± 1.4. The PSAS Score was 16.7 ± 8.6 and the OSAS was 13.5 ± 5.7.

The GS group had a significantly wider scar than the OC group (GS mean 21.8 ± 9.21 mm; OC mean 11.5 ± 8.2 mm; *p* = 0.034; *unpaired two-sided t test*). However, neither the length of the scar nor the cosmetic satisfaction (PSAS, OSAS) was significantly different between the two groups. Detailed data is shown in Table 6.

**Table 6:** Cosmetic satisfaction; all data are displayed as median and interquartile range unless otherwise specified.

AWD (*n* = 18)	PSAS	OSAS	*p* value (PSAS / OSAS)
Cosmetic satisfaction			
Umbilicus (yes=15, no=3)	15 (19), 14 (0)	12 (6), 21 (0)	0.824^*^ / 0.076^*^
Hardening of the scar (yes=5, no=13)	18 (12), 14 (16)	12 (15), 13 (6)	0.503^*^ / 0.775^*^
Additional scars (yes=6, no=12)	14.5 (3), 15.5 (23)	18 (9), 11 (4)	1.000^*^ / 0.003^*^
Visible stiches (yes=12, no=6)	15.5 (14), 10 (16)	13.5 (7), 10.5 (11)	0.250^*^/ 0.250^*^
Correlation Coefficient			
Length of the scar (mm)	0.137	0.685	0.589^†^ / **0.002**^†^
Width of the scar (mm)	−0.045	−0.123	0.861^†^ / 0.626^†^
BMI (kg/m^2^)	0.290	−0.016	0.243^†^ / 0.951^†^
Number of surgeries	0.217	0.568	0.387^†^ / 0.014^†^

	GS (*n* = 12)	OC (*n* = 6)	*p* value (GS / OC)
PSAS	17.8 ± 8.7 ^a^	14.7 ± 8.9 ^a^	0.493^**^
OSAS	12.9 ± 3.2 ^a^	14.7 ± 9.4 ^a^	0.673^**^
Length of the scar (mm)	37.5 (78)	25.0 (112)	0.335^*^
Width of the scar (mm)	21.8 ± 9.2	11.5 ± 8.2	0.034^**^

Statistically significant *p*-values are in bold.

^a^Mean ± standard deviation.

^*^Mann–Whitney-U; ^**^unpaired *t* test; ^†^ Spearman test

OSAS showed significant correlation with the length of the scar (r = 0.685, *p* = 0.002; *Spearmen test*), otherwise neither OSAS nor PSAS showed significant correlation with number of surgeries or the appearance of the scar (Table 6).

No statistically significant differences of GIQLI, cosmetic satisfaction, back pain, reflux, stool frequency or BSS could be found between GS and OC (data not shown).

## Discussion

The main findings of our pilot study were that patients born with AWDs had significantly decreased scores concerning motor abilities and gastrointestinal quality of life. Additionally, we present data of lung function, exercise performance, electromyography, stance and gait analyses, ultrasound of the abdominal wall as well as cosmetic satisfaction of 18 AWD patients at a mean follow-up of 13 years and compare the obtained values to a healthy matched control group.

GS is defined as complicated in the presence of concomitant intestinal atresia, volvulus, necrosis or perforation.^16–18^ The definition of giant omphalocele is more diverse and ranges from inability to achieve primary closure, different amount of liver in the sac (>50–75%) or size of the defect (>5 cm).^19,20^ For our study, we have defined a giant OC as a size of the defect greater or equal to 5 cm. Nevertheless, we did not find any differences in our outcome parameters between uncomplicated and complicated GS or small and giant OCs. However, these findings have to be interpreted with caution due to the limited number of patients included in the respective subgroups.

Spirometry did not reveal any significant long-term pulmonary impairment of AWD patients suggesting that the abdominal wall malformation per se does not necessarily influence the cardiopulmonary function. Only two patients had a restrictive ventilation disorder and these children were born with a giant OC associated with lung hypoplasia (compare Table 1, Patient ID 14 and 18). This finding is in line with previous studies showing that patients with giant OCs may suffer from long term respiratory difficulties.^32^

Cardiopulmonary performance capacity plays an important role in health and well-being.^33^ Therefore, cardiopulmonary exercise performance testing should be part of the long-term examinations of children with congenital malformations. While a reduced relative performance capacity has already been found for esophageal atresia and anorectal malformation patients,^12,13^ the cardiopulmonary performance has only been described once for patients with AWDs.^14^ Zaccara and coworkers examined 18 patients with large AWDs (GS > 4 cm, OC > 6 cm) who have completed a stress test consisting of running on a treadmill with a stepwise increase in workload until exhaustion. The authors measured time of exercise (TE) and maximal oxygen consumption (VO_2max_) during the whole exercise as well as heart rate and blood pressure at baseline (HR and BP) and the end of the exercise (HR_max_ and BP_max_). AWD patients reached HR_max_ after a significantly shorter TE and VO_2max_ was significantly lower compared to a healthy pediatric population. The authors, however, suggested that their findings may result from being unfit instead of the illness per se and concluded that further investigations are necessary. Our results did not show any differences in the studied parameters of cardiopulmonary exercise performance testing comparing AWD patients to healthy controls.

The Dordel-Koch-Test has been used in the literature before to assess the locomotor function in patients with congenital anomalies such as anorectal malformations.^12^ The AWD cohort had significantly higher values revealing worse locomotor function compared to the control group (compare Table 3). Especially the exercises “sit up” and “lateral jumping” which are related to core muscle activity revealed significantly worse scores. This might be related to a functional disability of the abdominal wall muscles. Nevertheless, the clinical relevance and impact of the numerical differences in real life remain unclear. The questions whether or not AWD patients may benefit from long-term follow-up combined with targeted physical therapy has to be answered in future studies.

Neither gait and stance analyses nor electromyography of the abdominal wall muscles have been performed in AWD patients before. Gait analysis is used for children with cerebral palsy and surface electromyography plays an important role for instance when diagnosing neuromuscular, urodynamic or laryngeal disorders.^34–38^ However, we could not find any significant differences suggesting that there are no long-term sequelae in muscle activation and innervation of abdominal wall muscles in AWD patients. Additionally, the sonographic thickness measurement of the abdominal wall revealed no significant difference between the AWD and control group confirming the hypothesis that the structure of the abdominal wall muscles develops normally in people born with an AWD. These two findings could be communicated to parents of affected patients in order to dispel concerns about long-term outcome regarding the muscular abdominal wall. A potential functional disability of the abdominal wall muscles as suggested by the differences in parts of the DKT has to be clarified in future studies.

QoL consists of social and physical well-being and might be affected by gastrointestinal disorders in patients born with an AWD. Previous studies have already assessed the quality of life of AWD patients using the pediatric quality of life inventory (PedQoL) and in one of these studies differences in young adults have been found.^11,39,40^ It has also been described that 25% of patients with AWD suffer from chronic abdominal complaints maybe due to intraabdominal adhesions.^40^ To the best of our knowledge, our study is first one to apply the GIQLI for patients with AWDs. In the AWD group, we found a statistically significant lower score of GIQLI. Therefore, gastrointestinal disorders may affect patients following AWD repair through child- and adulthood in daily life. These findings and their real-life impact, however, have to be re-examined in studies with a larger group of patients. Nevertheless, we did not find any significant differences between the AWD group and controls concerning stool frequency or consistency.

Cosmetic satisfaction is of pivotal importance for patients who have undergone AWD repair and additional abdominal surgery due to complications. Especially, the dissatisfaction because of an abnormal/lacking umbilicus is a major issue; almost half of AWD patients experience psychological stress if they do not have an umbilicus.^5,41^ In our population, however, more than two thirds of the patients (71%) rated the overall quality and appearance of their scar as quite good (PSAS overall opinion: 3.9 ± 2.8) and we found no significant correlation between an absent umbilicus and cosmetic satisfaction. However, cosmetic satisfaction may change over time and might be a concern with growing age. Umbilicus reconstruction is often performed at a higher age.^5,41–43^

A limitation of our study is the relatively small sample size of 18 patients. Additionally, 5 out of 18 children were not able to complete CPET due to their height and in 4 out of 18 it was not possible to detect muscle mass due to technical reasons and low weight of the patients. However, in orphan pediatric diseases it is difficult to obtain large sample sizes and therefore possible statistically significant difference may be missed due to the small sample size. An additional limitation is that AWDs are heterogenous diseases and therefore our findings observed in a relatively small number of patients with heterogenous diseases are confounded by a certain lack of power. Moreover, even though the values for DKT and GIQLI were statistically significantly different between AWD patients and controls, the impact of these difference for real life remains unclear. Strengths of this study, however, are a mean long-term follow-up of 13 years ranging from 7 to 18 years and the inclusion of an age-, sex-, BMI- and physical activity matched control group.

In conclusion, we present the feasibility of an extensive long-term follow-up examinations of AWD patients. We found decreased motor abilities and GIQLI scores while cardiopulmonary function was not different compared to healthy controls. However, due to the limited number of patients in our pilot study, larger ideally multicentric studies are mandatory in order to confirm our results.

### Supplementary Information

Supplementary Material

## Data Availability

The data that support the findings of this study are available from the corresponding author upon reasonable request.
