# Supplementary material for: Long-term motor activity, cardiopulmonary performance and quality of life in abdominal wall defect patients
Source: Pediatr Res. 2023 Dec 5;95(4):1101–9. doi: 10.1038/s41390-023-02900-y (PMC10920181; doi:10.1038/s41390-023-02900-y)

Supplementary Table 1: Anthropometric data, results of spirometry, spiroergometry, DKT and QoL of patients with gastroschisis and omphalocele. All data are displayed as mean ± standard deviation unless otherwise specified.

	Gastroschisis (n=12)	Omphalocele (n=6)	p-value
**Age**	11.9 ± 3.5	13.8 ± 3.4	0.287[a]
**Anthropometry**			
Height [m]	1.57 (0.28)[c]	1.69 (0.17)[c]	0.151[b]
Body Weight [kg]	45.7 ± 16.3	53.0 ± 18.1	0.403[a]
BMI	19.3 ± 4.7	19.5 ± 4.1	0.954[a]
Body Fat [%]	7.75 (19.0)[c]	17.5 (18.1)[c]	0.820[b]
Muscle Mass [kg/Height²][d]	6.3 ± 1.3	6.2 ± 1.4	0.874[a]
Number of surgeries	6.3 ± 1.3	6.2 ± 1.4	0.588[a]
Physical Activity Level[f]	2 (0)[c]	2 (1)[c]	0.616[b]
**Spirometry**			
VC_{max} [%]	2.8 ± 1	3.0 ± 0.9	0.765[a]
Tiffeneau Index [%]	86.6 ± 7.6	85.9 ± 3,9	0.781[a]
**Spiroergometry**	**(n=8[e])**	**(n=5[e])**	
Relative Performance [%]	93.6 ± 14.9	104.6 ± 36.0	0.546[a]
RER	1.14 ± 0.08	1.19 ± 0.07	0.261[a]
peak VO_2 [ml/kg/min]	42.1 (11)[c]	38.4 (17)[c]	0.717[b]
O_2/HR [ml]	10.6 (5)[c]	12.7 (4)[c]	0.796[b]
EQO_2	22.3 ± 3.0	20.9 ± 3.3	0.450[a]
BR FEV%	11.9 ± 10.2	8.4 ± 12.6	0.619[a]
**DKT**			
Mean DKT	3.6 (1.0)[c]	3.3 (1.0)[c]	0.820[b]
Lateral Jumping	4.0 (1.0)[c]	4.0 (2.0)[c]	1.000[b]
Sit and Reach	3.5 (2.0)[c]	4.5 (3.0)[c]	0.494[b]
Sit-Ups	4.0 (1.0)[c]	3.5 (2.0)[c]	0.291[b]
Long Stand Jump	5.0 (2.0)[c]	4.0 (1.0)[c]	0.385[b]
One-legged Stand	1.0 (3.0)[c]	2.0 (4.0) [c]	0.494[b]
Push-Ups	3.0 (1.0)[c]	2.0 (2.0)[c]	0.494[b]

**QoL**			
GIQLI	137.5 ± 7.7	136.5 ± 5.2	0.749[a]
Bristol Stool scale	3.5 ± 1.0[c]	4.0 ± 1.0[c]	0.053[a]
Cosmetic satisfaction	26.8 ± 5.8	28.0 ± 4.42	0.620[a]

m…meter; kg…kilogram; VC_{max}…maximum vital capacity; RER…Respiratory Exchange Ratio; peak VO_2…peak oxygen uptake; O_2/HR…oxygen pulse; EQO_2…respiratory equivalent for oxygen, BR…breathing reserve, FEV…Forced expiratory volume; DKT…Dordel Koch test; QoL…quality of life; GIQLI…Gastrointestinal Quality of Life Index

[a] unpaired t-test, [b] Mann-Whitney-U test, [c] median (IQR)

[d] n=14; it was not possible to detect muscle mass in 4 cases

[e] 5 patients were not able to perform spiroergometry because they were too short for ergometry

[f] Physical Activity Level (once per month=0, once per week=1 several times a week=2, daily=3)

Supplementary Table 2: Ultrasound data of gastroschisis and omphalocele patients. All data are displayed as mean ± standard deviation unless otherwise specified.

	**Gastroschisis (n=12)**	**Omphalocele (n=6)**	**p-value**
I OE	3.9 ± 1.7	3.6 ± 2.3	0.806[a]
II OE	3.1 ± 1.6	3.6 ± 1.6	0.571[a]
III OE	3.8 (2.2)[c]	4.1 (3.5)[c]	0.892[b]
IV OE	4.0 ± 1.6	4.4 ± 1.6	0.639[a]
V OE	6.1 ± 2.4	5.5 ± 2.4	0.630[a]
VI OE	6.0 ± 2.1	5.8 ± 2.4	0.845[a]
I OI	3.1 ± 1.1	3.0 ± 1.1	0.987[a]
II OI	3.5 ± 1.0	4.3 ± 1.8	0.327[a]
III OI	4.7 ± 1.5	6.2 ± 2.2	0.151[a]
IV OI	4.3 ± 1.5	6.4 ± 2.1	0.061[a]
V OI	6.3 ± 1.7	6.4 ± 2.1	0.929[a]
VI OI	6.8 ± 2.0	6.8 ± 2.0	0.973[a]
I TA	2.9 ± 0.8	2.3 ± 0.9	0.146[a]
II TA	3.0 (1.5)[c]	2.6 (0.6)[c]	0.961[b]
III TA	2.0 (2.3)[c]	2.4 (2.5)[c]	0.750[b]
IV TA	2.3 (1.4)[c]	3.4 (2.2)[c]	0.180[b]
V TA	3.6 ± 1.8	3.6 ± 1.5	0.983[a]
VI TA	3.7 ± 1.1	3.0 ± 1.1	0.226[a]
B_1 RA right	8.6 ± 2.7	7.8 ± 3.6	0.637[a]
B_1 RA left	8.8 ± 2.7	8.8 ± 4.1	0.942[a]
B_2 RA right	9.0 ± 2.7	8.8 ± 3.1	0.884[a]
B_2 RA left	9.3 ± 2.8	9.5 ± 3.2	0.892[a]
C RA right	8.8 ± 2.7	9.3 ± 3.4	0.778[a]
C RA left	8.7 ± 2.6	8.9 ± 3.5	0.901[a]

[a] unpaired t-test; [b] Mann-Whitney-U test; [c] median (IQR)

Supplementary Table 3: Ultrasound data of all AWD patients comparing thickness of left and right abdominal wall muscles. All data are displayed as mean ± standard deviation unless otherwise specified.

	**Right (n=18)**	**Left (n=18)**	**p-value**
I OE / II OE	2.7 (1.9)[c]	2.6 (1.5)[c]	0.270[b]
III OE / IV OE	3.5 (1.9)[c]	3.4 (2.1)[c]	0.654[b]
V OE / VI OE	5.8 (2.5)[c]	5.9 (3.3)[c]	0.561[b]
I OI / II OI	3.3 (2.0)[c]	3.2 (2.0)[c]	0.957[b]
III OI / IV OI	5.0 (2.0)[c]	4.7 (2.5)[c]	0.667[b]
V OI / VI OI	6.6 ± 1.7	6.6 ± 1.7	0.900[a]
I TA / II TA	2.8 ± 0.9	2.7 ± 0.8	0.578[a]
III TA / IV TA	2.5 (2.0)[c]	2.9 (1.5)[c]	0.471[b]
V TA / VI TA	3.5 ± 1.2	3.5 ± 1.0	0.163[a]
B_1 RA right / left	8.3 ± 2.4	8.5 ± 2.7	0.282[a]
B_2 RA right / left	8.8 ± 2.5	9.0 ± 2.5	0.278[a]
C RA right / left	9.0 ± 2.7	8.9 ± 2.6	0.339[a]

[a] paired t-test; [b] Wilcoxon test; [c] median (IQR)

Supplementary Figure 1: Placement of EMG electrodes.

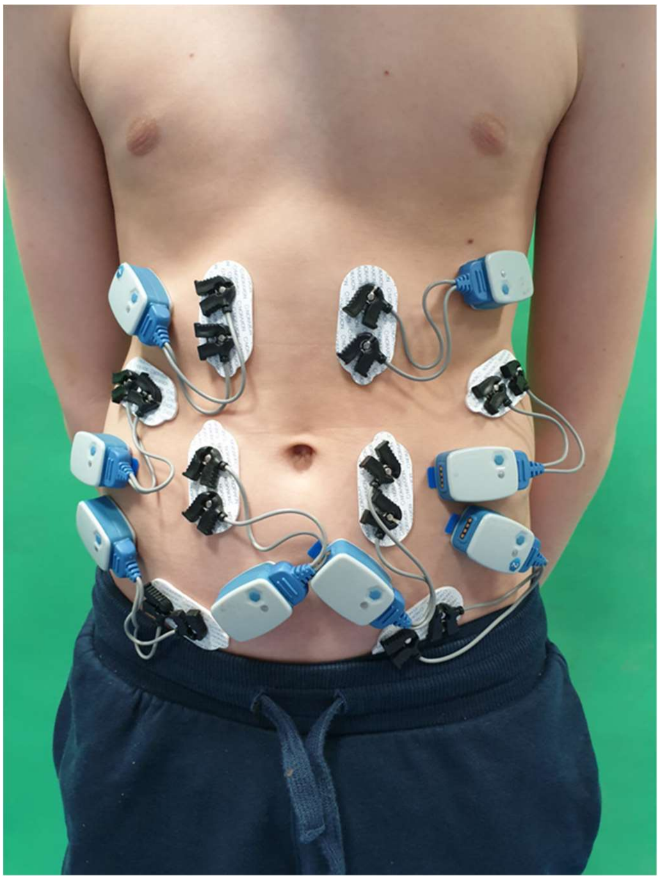

Supplementary Figure 2: Exercises to measure activity of the M. rectus abdominis (RA), M. obliquus externus (OE)/internus (OI) and M. transversus abdominis (TA): Abdominal crunch – hold for three seconds (a); plank for 30 seconds (b); Holding up legs for 30 seconds (c); Sit ups (d); maximum voluntary contraction RA (e); maximum voluntary contraction OI/OE (f).

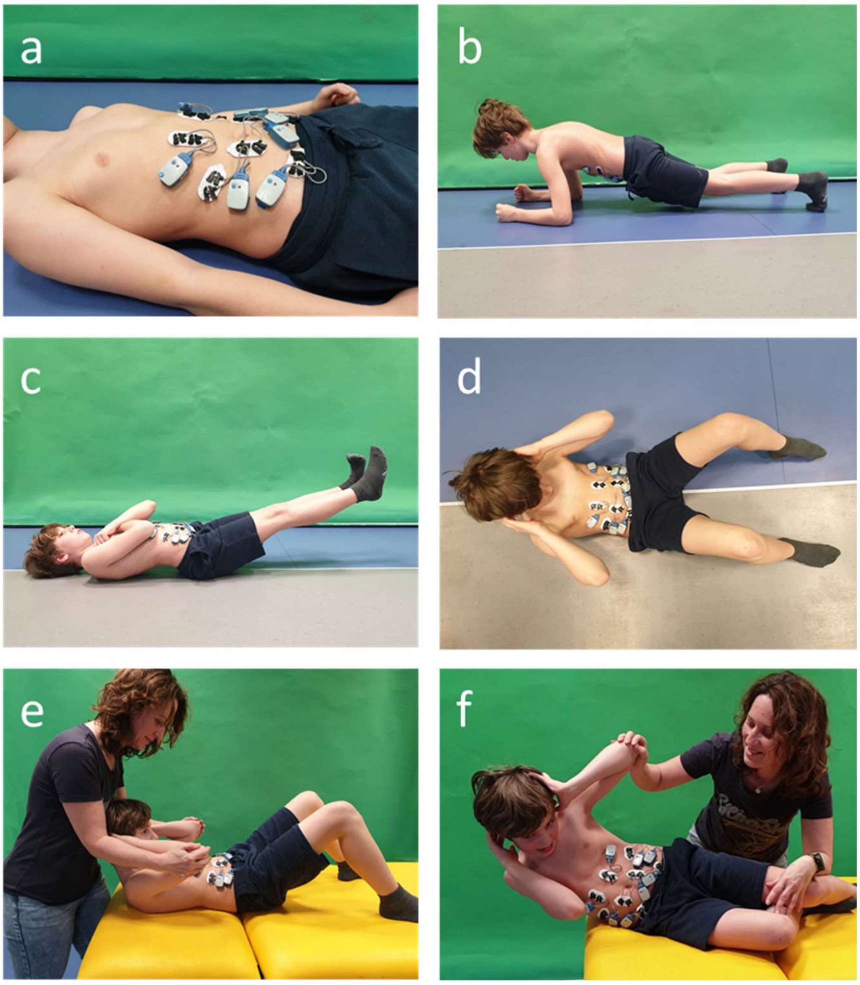

Supplementary Figure 3: Stance analyses: Ordinary relaxed stand (a) Matthias' Arm-Raising Test (b) Gait analyses: walking over the plate for 3 minutes (c)

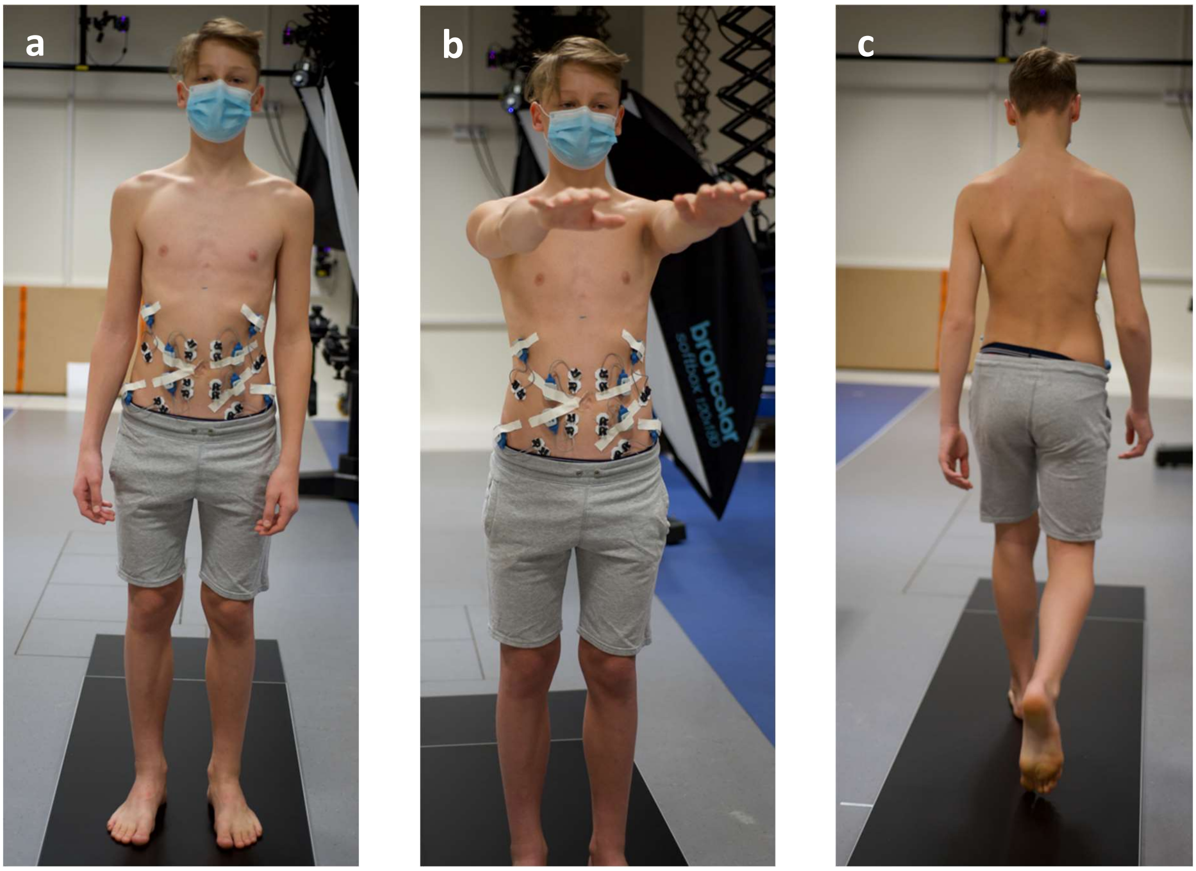

Supplement: Supplementary file 1 — Supplementary Material [file 41390_2023_2900_MOESM1_ESM.pdf]
